# Light-induced metal-like surface of silicon photonic waveguides

**DOI:** 10.1038/ncomms9182

**Published:** 2015-09-11

**Authors:** Stefano Grillanda, Francesco Morichetti

**Affiliations:** 1 Dipartimento di Elettronica, Informazione e Bioingegneria, Politecnico di Milano, 20133 Milano, Italy

## Abstract

The surface of a material may exhibit physical phenomena that do not occur in the bulk of the material itself. For this reason, the behaviour of nanoscale devices is expected to be conditioned, or even dominated, by the nature of their surface. Here, we show that in silicon photonic nanowaveguides, massive surface carrier generation is induced by light travelling in the waveguide, because of natural surface-state absorption at the core/cladding interface. At the typical light intensity used in linear applications, this effect makes the surface of the waveguide behave as a metal-like frame. A twofold impact is observed on the waveguide performance: the surface electric conductivity dominates over that of bulk silicon and an additional optical absorption mechanism arises, that we named surface free-carrier absorption. These results, applying to generic semiconductor photonic technologies, unveil the real picture of optical nanowaveguides that needs to be considered in the design of any integrated optoelectronic device.

## Abstract (web summary)

On the nanoscale materials exhibit surface phenomena that do not occur in the bulk. Here, Grillanda *et al*. demonstrate that the surface of silicon photonic waveguides change according to the intensity of the light propagating in the waveguide, underlining considerations in design of integrated optoelectronic devices.

## Graphical abstract

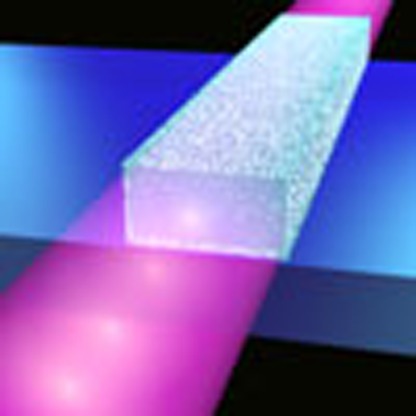

When the size of a device is reduced to the nanoscale, its behaviour may be strongly affected by physical effects localized at the surface^1^,^2^,^3^,^4^,^5^, where phenomena not occurring in the bulk of the material may arise^6^. For instance, in semiconductor nanomembranes^1^,^2^ and nanowires^3^,^4^,^5^, the impact of the surface can be so relevant that some properties, such as the electrical conductivity, are dominated by surface contributions^1^,^2^.

In photonics, surface effects have been traditionally associated to imperfections and roughness on the walls of integrated waveguides^7^,^8^. However, on the surface of semiconductor waveguides such as in silicon (Si), the natural termination of the crystal lattice results in a distortion of the energy bands^1^ and in the creation of intra-gap states^6^, that are localized in the forbidden gap and are responsible for the absorption of photons with energy lower than the bandgap of the bulk material^9^ (Fig. 1a). Surface-state absorption (SSA) is thus a single-photon process generating free carriers at the core/cladding interface. So far, SSA has been exploited only to develop photonic devices such as near-infrared all-Si photodetectors^10^,^11^,^12^,^13^,^14^ and all-optical Si modulators^15^. Actually, the impact of surface states on Si photonic waveguides is much broader and may become relevant in the behaviour of optoelectronic integrated devices, especially when their size approaches the nanoscale. The relevance of these issues has not been perceived yet because the effect of carrier generation induced by surface states on the electrical and optical properties of a waveguide has been neither quantified nor compared with bulk effects.

In this work we show that at typical light intensity the surface of Si photonic waveguides exhibits a metal-like behaviour. In fact, below the high intensity threshold where non-linear bulk effects become relevant, an intermediate regime exists where the electric conductivity of weakly doped (10^15^ cm^−3^) Si nanowaveguides is dominated by the carrier generation induced by SSA. The density of the free carriers generated on the surface is more than 100 times larger than that of the free carriers thermally generated in the bulk, thus making the surface properties move towards those of metals. This effect overwhelms the conductivity of bulk Si in the absence of light, and becomes relevant in Si electro-optic devices where slightly doped regions are used, such as in modulators^16^,^17^,^18^,^19^ and micro-heaters embedded in the waveguide core^14^. Furthermore, we provide the first direct evidence that surface free carriers are responsible for an additional optical absorption mechanism, hereinafter named surface free-carrier absorption (SCA), that increases linearly with light intensity. Our results reveal that in Si waveguides this is the largest loss contribution with linear dependence on optical power, being about one order of magnitude larger than two-photon absorption (TPA).

## Results

**Figure 1:**
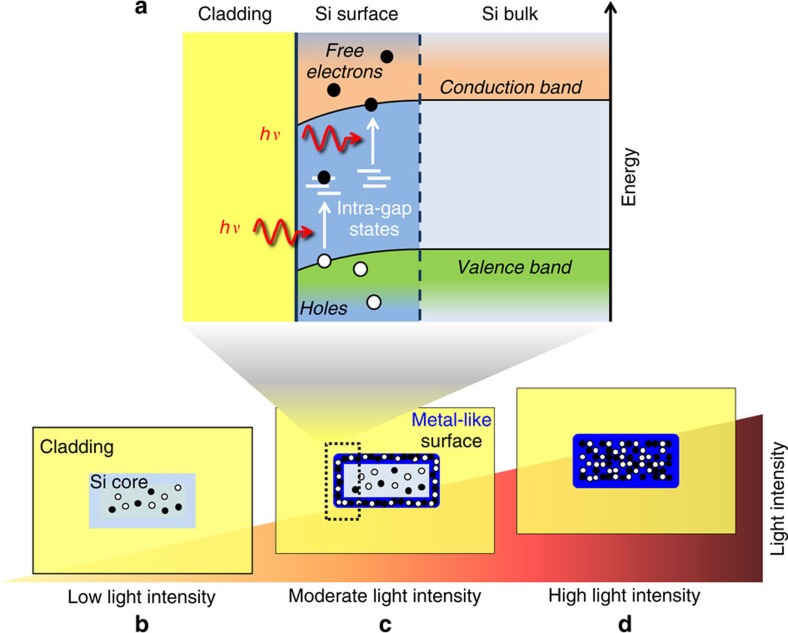
Metal-like behaviour at the surface of Si waveguides in presence of light. (**a**) Free carriers are generated at the waveguide surface because of intra-gap states (*hν* is the photon energy). In the electrical domain the Si core is a photoconductor whose behaviour can be divided into three domains under light injection: (**b**) low and (**d**) high light intensity where the conductivity of the waveguide is dominated by the carriers located in the bulk of the core, that are due to the doping (low intensity) and to TPA (high intensity); (**c**) an intermediate regime where surface free carriers largely dominate the waveguide photoconductivity and induce a metal-like surface.

The intermediate regime where the electric properties of the waveguide are dominated by SSA-induced carriers is schematically shown in Fig. 1. At low and high light intensity the waveguide conductivity *σ* is dominated by the carriers located in the bulk of the Si core, that are respectively due to the waveguide doping and to TPA (Fig. 1b,d). Vice versa, at moderate light intensity the carrier photogeneration occurring at the core/cladding interface is so massive (Fig. 1c) that the surface is filled with carriers, *σ* is essentially given by the conductivity of the surface, that exhibits a metal-like behaviour. In the following sections the existence of this metal-like surface is demonstrated by cross-relating the results of optical domain SCA measurements with electric domain measurements performed with a recently developed surface-state photodetector^10^.

### Light-induced absorption at the surface

**Figure 2:**
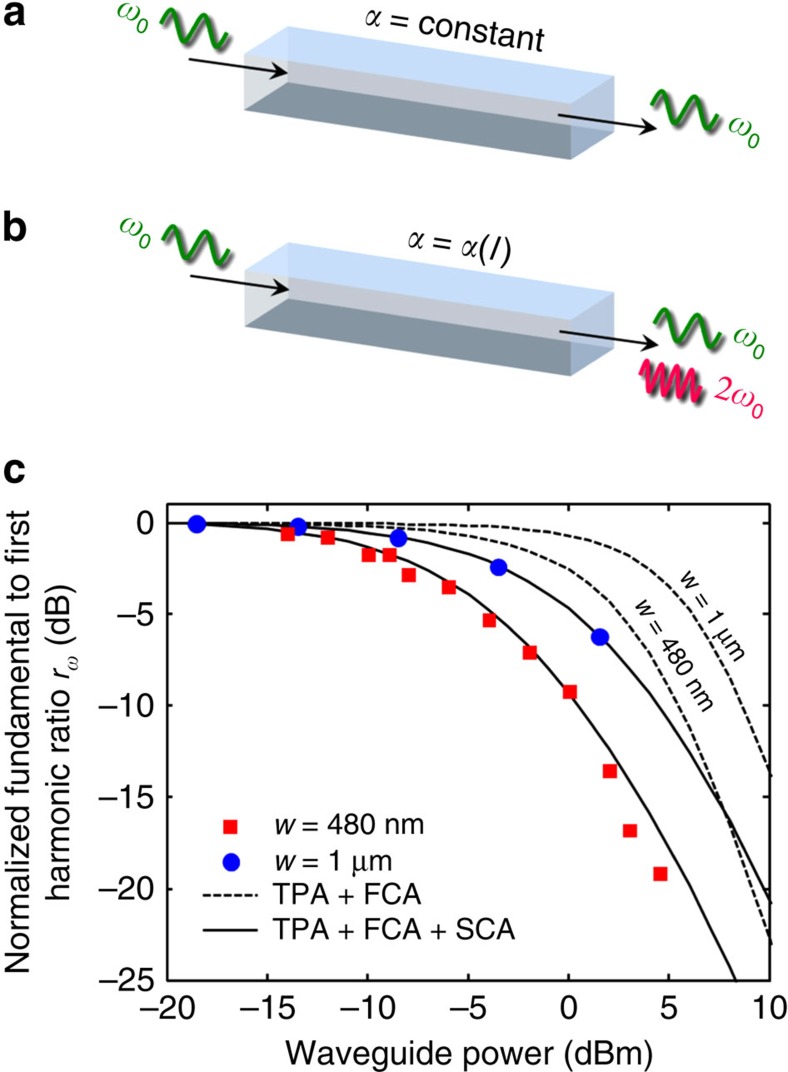
Observation of surface carrier absorption (SCA) in Si waveguides. (**a**–**b**) Experimental technique developed to measure SCA from the intensity of the harmonic components at the output of the waveguide when a weakly modulated signal (at angular frequency *ω*_0_) is injected at the input. (**c**) The measured ratio between the fundamental and first harmonic *r*_*ω*_=*I*(*ω*_0_)/*I*(2*ω*_0_) can be explained only with an absorption model that includes the effect of the waveguide surface (SCA). Two waveguide widths *w*, 480 nm and 1 μm, are considered.

The optical absorption due to surface free carriers was measured according to a novel technique whose principle is sketched in Fig. 2a,b. Let us assume that light with sinusoidal intensity modulation at angular frequency *ω*_0_ around the optical carrier is injected in a photonic waveguide with total propagation loss *α*. Ideally, if *α* were constant with light intensity *I*, the output would exhibit only a spectral component at *ω*_0_ (Fig. 2a). Vice versa, if the waveguide loss depends on *I* also other spectral components, at harmonic frequencies 2*ω*_0_, 3*ω*_0_ and so on, appear at the output of the waveguide (Fig. 2b). The latter is the real scenario experienced by Si photonic waveguides, where different intensity-dependent absorption mechanisms contribute to the overall loss of the waveguide.

The intensity of the harmonic components *I*(*ω*_*i*_) at the output of the waveguide depends on the magnitude of each absorption mechanism. We model the dependence of the waveguide loss on light intensity as


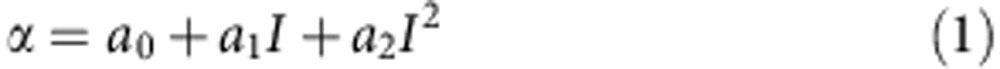


where *a*_0_, *a*_1_, *a*_2_ are coefficients. The first term of equation (1), *a*_0_, that does not depend on light intensity, represents the loss associated to SSA^9^ and to surface imperfections and roughness, that is the coupling of the optical mode with radiation modes (radiation loss) and with counter-propagating modes (backscattering)^7^,^8^. The second term of equation (1), *a*_1_*I*, that depends linearly on light intensity, includes the loss associated to TPA^20^, that occurs in the bulk of the waveguide core, and to SCA, that is due to the free carriers that fill the waveguide surface under light injection (see Supplementary Note 1 for details). The last term of equation (1), *a*_2_*I*^2^, that has a quadratic dependence on light intensity, represents the loss induced by the free carrier absorption (FCA) in the bulk of the waveguide core as a result of TPA mechanisms^20^. A summary of the absorption mechanisms and their dependence on light intensity is provided in Table 1.

By measuring the harmonic component *I*(*ω*_*i*_) at the waveguide output, quantitative information on the loss processes that occur can be found. This experimental technique is applied to channel waveguides fabricated in Si-on-insulator technology (doping 10^15^ cm^−3^). The rectangular Si core is buried in a glass cladding, has height of 220 nm and width *w*. No roughness smoothing treatment is performed at the core/cladding interface (see Methods section for details). All the measurements are performed for quasi-transverse electric polarized light at the wavelength of 1,550 nm. The modulation at *ω*_0_ is applied to the light at the input of the waveguide by means of an external intensity modulator, while the spectral components at the chip output are measured with a photodetector and an electrical-spectrum-analyzer (see Methods section, Supplementary Fig. 1 and Supplementary Note 2 for details).

Figure 2c shows the normalized ratio between the intensity of the fundamental and the first harmonic *r*_*ω*_=*I*(*ω*_0_)/*I*(2*ω*_0_) measured as a function of the optical power propagating in waveguides with *w*=480 nm (red squares) and *w*=1 μm (blue circles); waveguides length is, respectively, 7 mm and 9 mm. According to this technique, the loss coefficient *a*_0_, that is constant with light intensity, does not affect the ratio between the harmonic components, and therefore is not included in the analysis of the loss contributions. On the contrary, *r*_*ω*_ depends on *a*_1_ and *a*_2_, with slope that increases for larger absorption coefficients. Dashed lines show *r*_*ω*_ calculated under the assumption that absorption of light occurs only in the bulk of the waveguide core (TPA and FCA only). A waveguide TPA coefficient of *β*_TPA_=0.7 cm GW^−1^, that we measured in ref. 21 for this waveguide technology, has been used to evaluate *a*_1_ and *a*_2_. For the waveguide with *w*=480 nm (*w*=1 μm), at the optical power of 0 dBm, this results in a total loss due to TPA of about 6.5 × 10^−3^ dB cm^−1^ (3.2 × 10^−3^ dB cm^−1^), and in a loss due to FCA of about 1.3 × 10^−3^ dB cm^−1^ (8 × 10^−4^ dB cm^−1^) according to the equations of Soref and Bennett^22^. It is worth noting that provided *β*_TPA_ both the loss due to TPA and to FCA are given. However, this does not match the measured *r*_*ω*_ that for both waveguides deviates from the reference level (0 dB) at lower waveguide power and has a different slope. The observed behaviour can be explained only with a much larger *a*_1_ while keeping the same value of *a*_2_ (solid line), that means that a higher linear absorption coefficient is required without resulting in a higher quadratic absorption coefficient as well. Therefore, this cannot be explained with a larger *β*_TPA_, because an increase of the TPA induced loss would result in an increase of the FCA loss as well. Indeed this is the contribution of SCA, that is the loss induced by the free carriers that populate the waveguide surface.

**Figure 3:**
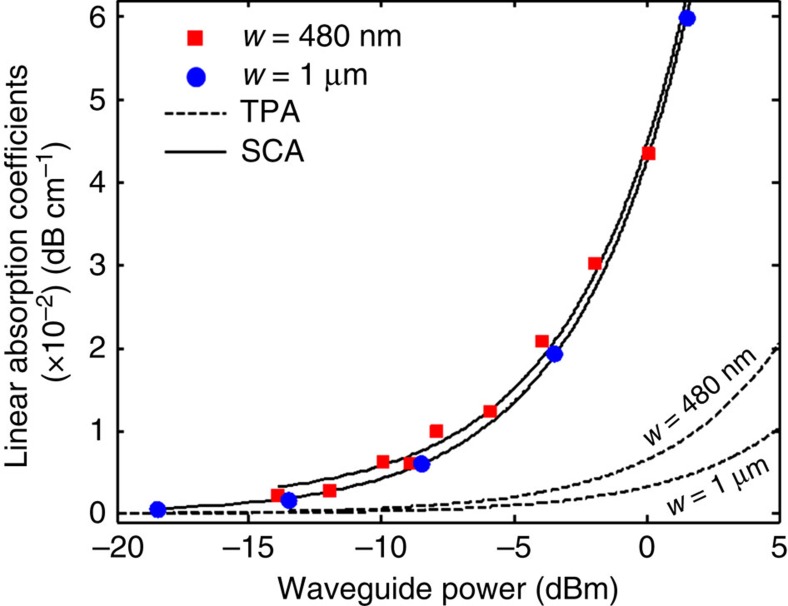
Absorption contributions with linear dependence on light intensity. The sum of SCA and TPA provides the linear loss term *a*_1_*I*. The surface carrier absorption, that is measured with the technique shown in Fig. 2, is about one order of magnitude larger than TPA, and therefore is the main source of optical loss with linear dependence on optical power.

Figure 3 reports the absorption contributions with linear dependence on light intensity, that are SCA and TPA, as a function of the optical power for the two waveguides (shown also in Supplementary Fig. 2). Red squares and blue circles indicate the experimental points derived from the measurement of Fig. 2c, whereas solid lines show the linear fit. At 0 dBm, the loss due to SCA amounts to about 4.5 × 10^−2^ dB cm^−1^ and 4.3 × 10^−2^ dB cm^−1^, respectively, for the waveguide with *w*=480 nm and *w*=1 μm. Therefore, the absorption induced by the surface free carriers at the core/cladding interface is about one order of magnitude larger than the TPA in the bulk (dashed lines). This means that in our Si photonic waveguides SCA is essentially the main source of optical loss with linear dependence on light intensity. At this power level, SCA is also higher than the loss associated to the doping, that is expected to be about 0.03 dB cm^−1^ for a 10^15^ cm^−3^ p-type Si waveguide.

Actually it is worth noting that, as shown in Fig. 3, the loss induced by SCA is similar for the two considered waveguides, that have different widths. This is because the integral of the light intensity of the modes on the two waveguides along the waveguide surface (where surface states are located) is similar, as confirmed by electromagnetic simulations. This results in a comparable local density of surface carriers, and therefore in about the same SCA. Also, it is worth considering that the technique used to measure SCA is neither dependent on the material system employed for the waveguide fabrication nor on its geometry, and therefore can be directly applied to any photonic waveguide technology.

### Light-induced conductivity at the surface

**Figure 4:**
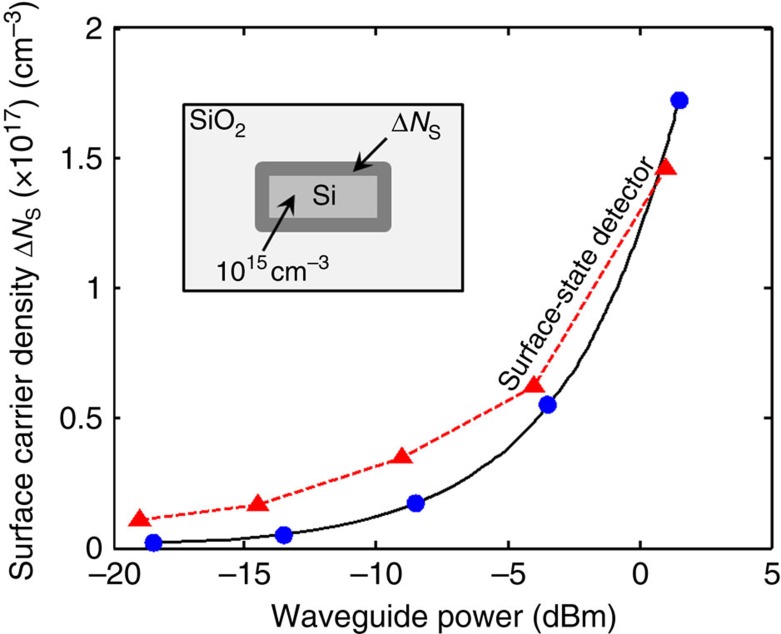
Light-induced surface carrier density in Si waveguides. Density of free carriers Δ*N*_s_ located on the surface of the 1-μm wide waveguide at the core/cladding interface (indicated with dark grey in the inset). The carrier density obtained from the optical loss measurement of Figs 2 and 3 (blue circles) is in good agreement with that provided in the electrical domain by a surface-state photodetector (red triangles).

We now evaluate the effects of the surface free carriers in the electrical domain. Figure 4 shows the density Δ*N*_s_ of free carriers located on the surface of the waveguide with *w*=1 μm as a function of the optical power (blue circles). This carrier density is calculated from the measured SCA induced loss of Fig. 3 using the equations given in ref. 22 and assuming that surface states are located within the first three/four atomic layers (∼1 nm) from the Si surface^23^. Although the waveguide is excited with moderate optical power, the photogeneration of free carriers on its surface is massive. In fact, only −18 dBm are sufficient to fill the surface with twice as much the carriers available in the bulk of the waveguide in absence of light (∼10^15^ cm^−3^). Then, Δ*N*_s_ grows rapidly with light intensity, amounting to more than 10^17^ cm^−3^ at 0 dBm, that is respectively two and four order magnitudes larger than the number of carriers located in the waveguide bulk in absence of light and induced by TPA (∼10^13^ cm^−3^). This means that a 1 nm thick highly conductive frame is created by the light at the border of the Si core, as shown in Fig. 1c.

Furthermore, we compare the density of surface carriers calculated from the loss measurement of Figs 2 and 3 with that measured on the same waveguide with a surface-state photodetector (red triangles in Fig. 4) that we developed. This detector, whose operation principle is detailed in ref. 10, provides in the electrical domain the density of free carriers located on the waveguide surface. Good agreement across the entire power range is found between Δ*N*_s_ measured in the optical (blue circles) and electrical (red triangles) domain, thus confirming the result achieved with the approach presented in this work. Similar results were found for the 480 nm wide waveguide (Supplementary Fig. 3).

**Figure 5:**
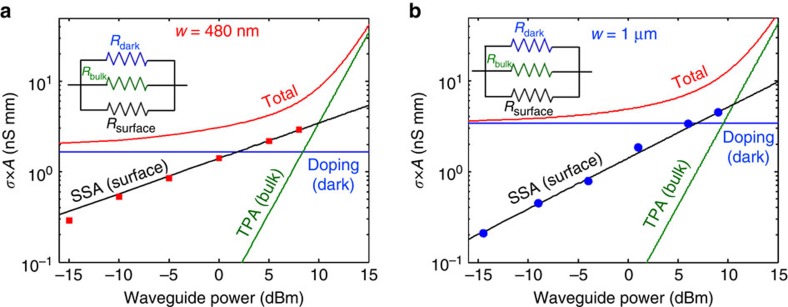
Behaviour of Si waveguides in the electrical domain. Conductivity per unit of area *σ* × *A* as a function of the optical power for waveguides with (**a**) *w*=480 nm and (**b**) *w*=1 μm. At low and high light intensity the photoconductivity of the waveguide is given essentially by the carriers located in the bulk of the core (respectively due to the doping and to TPA). At moderate light intensity the effect of the surface emerges and the waveguide conductivity is dominated by the surface carriers. The insets show the model of the Si core of the waveguide in the electrical domain.

Finally, we investigate the effect of the photogenerated surface carriers on the overall electrical conductivity *σ* of the waveguide. Figure 5a shows the contribution to *σ* of the electrical carriers located in the Si core as a function of the optical power for the waveguide with *w*=480 nm. Both the contributions of the surface (red squares) and of the bulk of the waveguide are shown (blue line for the doping and green line for TPA). For each physical process that contributes to *σ*, the area *A* in which it occurs has been taken into account, so that in Fig. 5a we show *σ* × *A*. This means that the contribution of the surface carriers is integrated only on the surface of the waveguide, whereas those related to the doping and to TPA are integrated on the whole core area. The red line indicates the total conductivity of the waveguide, that is the sum of the contributions due to doping, TPA and SSA. From this analysis, an electrical model of the Si waveguide emerges, that is shown in the inset of Fig. 5a. In the electrical domain, each contribution to the electrical conductivity can be represented as a resistance, so that under light injection the total resistance *R* of the waveguide is given by the following relation^1^


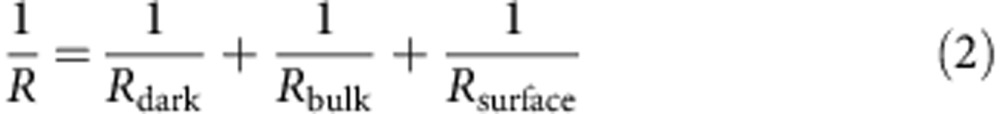


where *R*_dark_, *R*_bulk_ and *R*_surface_ are respectively the resistances due to the doping in absence of light, to the bulk (TPA) and to the surface (SSA). At low light intensity (<2 dBm), the doping contribution dominates and *σ* is essentially given by the carriers thermally generated in the bulk of the waveguide core; in this regime, *R* is well approximated by *R*_dark_. The bulk conductivity dominates also at high intensity (>10 dBm), where free carriers are generated by TPA and *R* is mainly given by *R*_bulk_. In contrast, at moderate light intensity (in the range 2–10 dBm) the effect of the surface emerges, and the main responsible to the waveguide photoconductivity are the surface free carriers. Therefore, in this regime the contribution of *R*_surface_ to the total resistance *R* must be taken into consideration. For instance, at a waveguide power of 8.5 dBm, where the doping and TPA equally contribute to the waveguide conductivity (1.65 nS mm, cross point between blue and green lines in Fig. 5a), the surface conductivity is about 3 nS mm, that is twice as large as the bulk conductivity.

The relevance of surface conductivity effects depends on the size of the waveguide. In fact, in a larger waveguide the conductivity due to the doping is higher, and therefore the light-induced surface conductivity becomes less relevant, as shown in Fig. 5b for the 1 μm wide waveguide. Comparing the two waveguide structures considered in this work, it is worth noting that the impact of the surface carriers on the photoconductivity can be significantly different even though the surface carrier absorption is almost the same (Fig. 3).

Finally, another consequence of the SSA-generated free carriers is the existence of a non-zero minimum conductivity for Si waveguides. As shown with black lines in Fig. 5, in presence of light the waveguide would exhibit a residual conductivity even in case the doping level were ideally reduced to zero.

## Discussion

Results presented in this work demonstrate that the surface of Si photonic waveguides significantly changes its nature according to the intensity of the light propagating in the waveguide.

At intensities that are typically used in linear applications (<10 dBm), a massive surface carrier generation occurs, that makes the surface appear as a metal-like frame, where the free-carrier density is more than two orders of magnitude higher than in the bulk of the waveguide. The creation of this quasi-metal layer, that has been so far ignored, is responsible for two main effects: (i) at moderate light intensity, for instance between about 0 and 10 dBm for single mode waveguides, the electrical properties of the waveguide are dominated by the surface free carriers, that largely overcome the conductivity of the bulk of the waveguide. This effect poses the ultimate limit to the electric conductivity of Si nanowaveguides; (ii) an additional optical absorption mechanism is induced, that we named SCA and has a linear dependence on light intensity, as for TPA. In conventional channel Si nanowaveguides SCA can be higher than the loss associated to the doping, and even more than ten times stronger than TPA. Therefore, the loss contributions due to surface states is expected to become dominant in waveguides where roughness induced scattering loss is reduced^24^. This unveils a new picture of optical Si waveguides, according to which a realistic description cannot be given without taking into consideration surface effects at the core/cladding interface.

The possibility to measure the optical and electric properties associated with the surface of Si waveguides enables also to access the necessary information to optimize the waveguide design, to either enhance or inhibit surface effects compared with the properties of the bulk of the waveguide. For example, surface effects are likely to play an important role in Si photonic modulators, based for instance on p-i-n junctions^16^ or capacitor structures embedded in Si waveguides^17^, as well as integrating electro-optic organic^18^ or 2D^19^ cladding materials. In these devices, the waveguide core is typically contacted to the metal electrodes through Si layers, which are slightly doped to avoid optical loss, but introduce a series resistance that limits the maximum modulation speed. As the optical field overlaps with these Si connections, a light dependent resistance associated with the generation of surface free carriers has to be considered to optimize the device design.

The relevance of SSA-induced carrier generation is as much pronounced as the waveguide size is reduced. Maximum impact is expected when the optical confinement is pushed so deeply into the subwavelength scale that the effective waveguide size approaches that of its own surface: this is for instance the case of nanoplasmonic structures, where the electric field is strongly localized and dramatically enhanced at a metal-Si interface^25^. In this case, a massive density of free carriers is expected to be generated by SSA well below the intensity threshold of non-linear effects.

These considerations are not limited to Si photonics, but hold for any optical waveguide technology where surface phenomena are likely to appear, such as indium phosphide^26^, germanium^27^, gallium arsenide^28^,^29^ and their compounds. The relevance of surface carrier generation processes is expected to depend on the energy gap of the core material compared with the wavelength of the light radiation and on the nature of the core/cladding interfaces. Therefore, the choice of the material used as waveguide cover layer and the quality itself of the surface, which is related to the fabrication processes, strongly affect the optical and electrical phenomena that occur at the waveguide surface.

## Methods

### Waveguides fabrication

Silicon photonic waveguides were fabricated from a commercial Si-on-insulator wafer with a 220-nm thick Si layer on a 2-μm thick oxide buffer layer. The waveguide pattern was written on a hydrogen silsesquioxane resist through electron-beam lithography and then transferred to the Si core by an inductively coupled plasma etching process according to the procedure described in ref. 30. The waveguide core is buried under a 1-μm thick cover layer, consisting of 550 nm of spun and baked hydrogen silsesquioxane and 450 nm of SiO_2_ grown by plasma enhanced chemical vapour deposition.

### Experimental setup

The light signal at the input of the waveguide is generated by means of a continuous-wave laser at 1,550 nm. A lithium niobate intensity modulator applies a weak sinusoidal modulation at frequency 500 kHz to the signal. An erbium-doped-fiber-amplifier and a variable-optical-attenuator are used to accurately control the light intensity at the input of the waveguide. Polarization controllers at the input and at the output of the modulator enable to control the polarization of the light respectively injected in the modulator and coupled to the Si photonic waveguide. At the output of the waveguide the light is collected by a photodetector, whose output feeds an electrical-spectrum-analyzer that enables to measure the harmonic components at the relevant frequencies. A schematic of the experimental setup can be found in Supplementary Fig. 1, additional details in Supplementary Note 2.

## Additional information

**How to cite this article:** Grillanda, S. *et al*. Light-induced metal-like surface of silicon photonic waveguides. *Nat. Commun.* 6:8182 doi: 10.1038/ncomms9182 (2015).

## Supplementary Material

Supplementary Information Supplementary Figure 1-3, Supplementary Notes 1-2 and Supplementary References.

## Figures and Tables

**Table 1:** Absorption mechanisms in Si photonic waveguides.

Waveguide absorption	*a*_0_	*a*_1_	*a*_2_
Surface (single-photon)	SSA	SCA	—
Bulk (two-photon)	—	TPA	FCA

Absorption mechanisms occurring in a Si waveguide and their dependence on light intensity according to equation (1). Single-photon and two-photon refer to the nature of the absorption mechanisms, respectively SSA (surface) and TPA (bulk), responsible for free carrier generation.
