# Supplementary material for: Light-induced metal-like surface of silicon photonic waveguides
Source: Nat Commun. 2015 Sep 11;6:8182. doi: 10.1038/ncomms9182 (PMC4579594; doi:10.1038/ncomms9182)
Supplement: Supplementary Information — Supplementary Figure 1-3, Supplementary Notes 1-2 and Supplementary References. [file ncomms9182-s1.pdf]

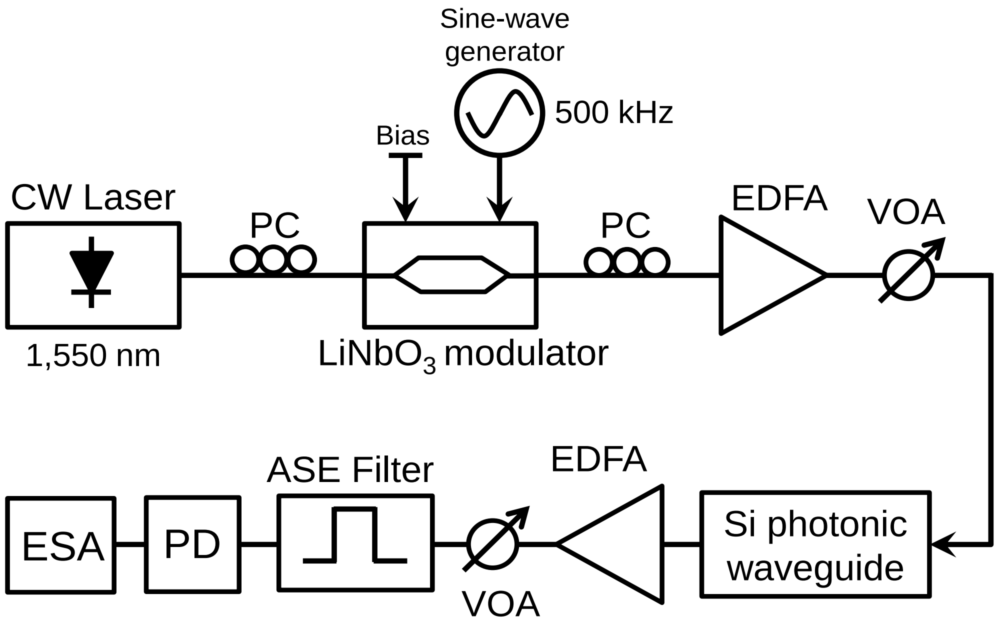

Supplementary Fig. 1 | Experimental setup. Schematic of the experimental setup utilized in this work for the measurement of the light-induced surface carrier absorption.

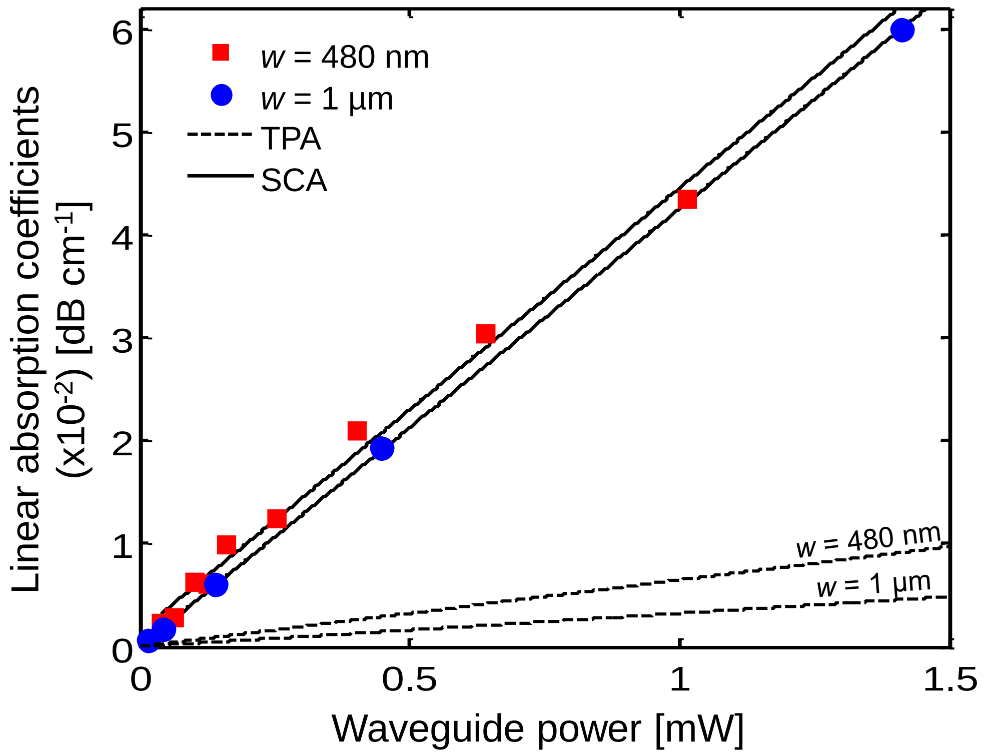

Supplementary Fig. 2 | Absorption contributions with linear dependence on light intensity (horizontal linear scale). The sum of SCA and TPA provides the loss term $a_1 I$. The surface carrier absorption, that is measured with the technique shown in Fig. 2 of the main manuscript, is about one order of magnitude larger than TPA, and therefore is the main source of optical loss with linear dependence on optical power. This figure reports the same data as Fig. 3 of the main manuscript with a horizontal mW-scale that well shows the linearity on optical power of the considered absorption contributions.

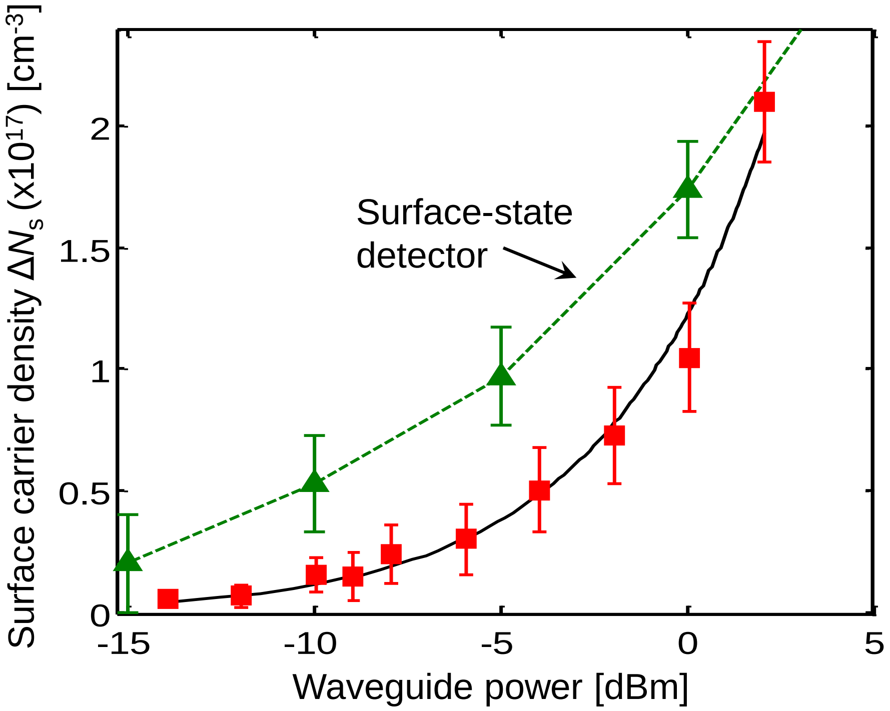

Supplementary Fig. 3 | Light-induced surface carrier density in a single-mode Si waveguide. Density of free carriers ΔN_s located on the surface of the 480 nm wide waveguide at the core/cladding interface. The carrier density obtained from the optical loss measurement of Fig. 3 of the main text (red squares) is in good agreement with that provided in the electrical domain by a surface-state photodetector (green triangles). Error bars have been added to show the accuracy of the two measurements. For the optical domain measurement (red squares) the accuracy is related to the sensitivity of the harmonic ratio versus the waveguide power [Fig. 2(c) of the main text], thus making the error bar increase for higher optical intensity. For the electrical domain measurement (green triangles) the error bars are related to the accuracy of the surface-state photodetector that we employed (see Ref. 10 of the main text).

Supplementary Note 1: Model of free carrier generation at the surface

Here we describe the model of free carrier generation induced by single-photon absorption processes, that is surface-state absorption (SSA), at the core/cladding interface of the waveguide. This model is described in complete analogy to that of two-photon absorption (TPA) [Ref. 20 of the main text], that governs the free carrier generation in the bulk of the waveguide.

The temporal behavior of the surface free carriers can be described with the following equation

$$\frac{\partial N_{\mathrm{s}}}{\partial t} = \frac{\alpha_{\mathrm{SSA}}}{h\nu} I - \frac{N_{\mathrm{s}}}{\tau_{\mathrm{s}}} \qquad (1)$$

where N_{s} is the density of free carriers on the surface of the waveguide, t is time, α_{SSA} is the loss due to SSA, $h\nu$ is the photon energy, I is light intensity and τ_{s} is the recombination time. The first term of Eq. 1 takes into account the photogeneration process of the carriers, whereas the second one their recombination, so that at the equilibrium the average density of free carriers on the waveguide surface is

$$N_{\mathrm{s}} = \frac{\alpha_{\mathrm{SSA}}\tau_{\mathrm{s}}}{h\nu} I. \qquad (2)$$

Therefore, since α_{SSA} is constant with light intensity, the density of photogenerated carriers on the waveguide surface increase linearly with light intensity.

Furthermore, considering that according to the theory of Drude [Ref. 20 of the main text and Supplementary Reference 1] the loss induced by the presence of free carriers increases linearly with their density, the surface free carrier absorption (SCA) has a linear dependence on light intensity.

Supplementary Note 2: Description of the experimental setup

Supplementary Figure 1 shows a schematic of the experimental setup that has been utilized to perform the experiments reported in this work.

The light signal at the input of the Si photonic waveguides is generated by means of a continuous-wave (CW) laser at the wavelength of 1,550 nm. An external lithium niobate ($LiNbO_3$) intensity modulator in Mach-Zehnder configuration is employed to apply a weak sinusoidal modulation at frequency 500 kHz to the optical signal. An erbium-doped-fiber-amplifier (EDFA) and a variable-optical-attenuator (VOA) enable to accurately and finely control the light intensity that is coupled to the waveguide. Polarization controllers (PCs) are utilized at the input and at the output of the modulator to control the polarization of the light respectively injected in the modulator and coupled to the photonic waveguide.

Lensed fibers with spot diameter of 1.7 μm are used to inject and collect the light to and from the Si photonic chip. The temperature of the optical chip is controlled within 0.1 K by a Peltier thermocooler integrated inside a customized holder.

At the chip output the light is collected by a photodetector (PD), whose output feeds an electrical-spectrum-analyzer (ESA) that enables to measure the harmonic components at the required frequencies. EDFA and VOA are here employed to provide constant average power to the PD. An amplified spontaneous emission (ASE) filter with bandwidth 0.25 nm is used to reduce the noise components introduced by the EDFAs included in the experimental setup.

Finally, it is worth noting that since this technique provides a direct loss measurement, thermal effects do not affect the measurement itself. In fact, thermal variations occurring on the sub-MHz time scale of this measurement would result in a variation of the phase of the optical field only, with no effects on the measured loss coefficients. Furthermore, as the modulation frequency is well below the free carrier recombination rate (which is in the ns time scale or faster), the proposed experiment provides information on the steady-state properties of the waveguide. Therefore, the free carrier dynamics has no impact on the measurement.

Supplementary References

[1] Li, S. S. Semiconductor Physical Electronics, (Springer New York, 2006).
